# The effect of percutaneouS vs. cutdoWn accEss in patients after Endovascular aorTic repair (SWEET): Study protocol for a single-blind, single-center, randomized controlled trial

**DOI:** 10.3389/fcvm.2022.966251

**Published:** 2022-08-19

**Authors:** Yuhang Zhou, Jiarong Wang, Jichun Zhao, Ding Yuan, Chengxin Weng, Tiehao Wang, Bin Huang

**Affiliations:** ^1^Department of Vascular Surgery, West China Hospital, Sichuan University, Chengdu, China; ^2^West China School of Medicine, West China Hospital, Sichuan University, Chengdu, China

**Keywords:** percutaneous, cutdown, access, endovascular aortic repair, randomized controlled trial

## Abstract

**Background:**

Endovascular abdominal aortic repair (EVAR) and thoracic endovascular aortic repair (TEVAR) have become the first-line treatment for aortic diseases, but current evidence is uncertain regarding whether a percutaneous approach has better outcomes than cutdown access, especially for patient-centered outcomes (PCOs). This study is designed to compare these outcomes of percutaneous access vs. cutdown access after endovascular aortic repair.

**Method:**

The SWEET study is a randomized, controlled, single-blind, single-center non-inferiority trial with two parallel groups in two cohorts respectively. After eligibility screening, subjects who meet the inclusion criteria will be divided into Cohort EVAR or Cohort TEVAR according to clinic interviews. And then participants in two cohorts will be randomly allocated to either intervention groups receiving percutaneous access endovascular repair or controlled groups receiving cutdown access endovascular repair separately. Primary clinician-reported outcome (ClinRO) is access-related complication, and primary patient-centered outcome (PCO) is time back to normal life. Follow-up will be conducted at 2 weeks, 1 month, 3 months postoperatively.

**Discussion:**

The choice of either percutaneous or cutdown access may not greatly affect the success of EVAR or TEVAR procedures, but can influence the quality of life and patient-centered experience. Given the very low evidence for ClinROs and few data for PCOs, comparison of the percutaneous vs. cutdown access EVAR and TEVAR is essential for both patient-centered care and clinical decision making in endovascular aortic repair.

**Trial registration:**

Chinese Clinical Trial Registry ChiCTR2100053161 (registered on 13th November, 2021).

## Introduction

Aortic diseases consist of degenerative, inflammatory, traumatic, infectious and congenital disorders, among which aortic aneurysm and aortic dissection are two common and life-threatening diseases. In terms of abdominal aortic aneurysm alone, the worldwide prevalence in people aged 75 to 79 was 2,275 per 100,000 in 2010 (1). Alarmingly, 34% patients died before reaching a hospital or during first admission once aneurysm ruptured (2). As for aortic dissection, the pre-hospital and in-hospital mortality was even higher, and reached 39% (2). Therefore, timely and correct intervention is quite essential. According to the European Society for Vascular Surgery (ESVS) 2019 guidelines for abdominal aortic aneurysm and 2017 guidelines for thoracic aortic diseases, endovascular abdominal aortic repair (EVAR) or thoracic endovascular aortic repair (TEVAR) is the first-line treatment option for aortic diseases when the anatomy is appropriate, and this recommendation is Class I with level of evidence A (3, 4).

Endovascular intervention techniques continue to evolve, and it is now feasible to obtain percutaneous femoral artery access and close the arterial puncture site with a vascular closure device remotely. Compared with conventional cutdown access, percutaneous procedure was reported to have potential advantages, involving lower risks of access site infection and lymphorrhagia, as well as shorter operation time (5). However, endovascular aortic repairs usually require large-profile sheath in the femoral arteries, which can carry challenges to percutaneous access closure, especially in patients with calcified or small femoral arteries (6, 7). Failure in percutaneous closure can lead to surgical repair. The risk-benefit balance in the choice of access procedures is still uncertain.

Most previous data were mainly based on cohort studies of different periods with low level of evidence (8). Only four randomized controlled trials (RCTs) were published comparing two types of access during EVAR, but all trials were judged to have low or very low certainty of evidence with high risk of bias (9–12). In addition, no RCTs have been published comparing two types of access in TEVAR. Though the recent meta-analysis revealed comparable access complication rates between two access procedures, it is noteworthy that no studies reported patient-centered outcomes (PCOs), for instance, patient's experience and quality of life after surgery (13). Considering its relatively small impact on prognosis and large impact on quality of life, PCOs may be a new perspective to shed light on the selection of access procedures in EVAR or TEVAR.

Given the current gap in evidence, this study intends to design a single-center, parallel, non-inferiority, randomized controlled trial in a 1:1 ratio, comparing both clinician-reported outcomes (ClinROs) and PCOs between percutaneouS vs. cutdoWn accEss in patients after Endovascular aorTic repair (SWEET) in two cohorts (EVAR and TEVAR).

## Materials and methods

### Study setting

The SWEET trial is a single-center study conducted at West China Hospital, Sichuan University in Chengdu, China. The protocol is reported in accordance with the Standard Protocol Items: Recommendations for Interventional Trials (SPIRIT) guidelines (14). The SPIRIT checklist is shown in Supplementary Table S1 (Supplementary Material). The trial was registered in the Chinese Clinical Trial Registry (registration number: ChiCTR2100053161) on 13th November, 2021.

### Participants

#### Recruitment

The participant characteristics derived from the PICO framework are presented in Supplementary Table S2 (Supplementary Material). Eligible patients with an indication for endovascular aortic repair will be informed concisely about the study by the attending resident. Subsequently, a trained research nurse will provide the consent and inform the patient in detail prior to the procedure. After careful consideration by the patient and relatives, the informed consent form will be signed prior to randomization in case of participation. Withdrawal from the study is permitted at any time for any reason, and it will not cause any consequence. To encourage participation, we will provide priority or fast track for outpatient appointments, follow-up assessment and consultation. If extra transportation costs are incurred due to the study, they will be paid by the sponsor.

#### Eligibility criteria

The study population consists of two independent cohorts: Cohort EVAR and TEVAR. The inclusion criteria of participants in Cohort EVAR are as follows: a. patient scheduled for EVAR because of abdominal aorto-iliac artery aneurysm or dissection, b. patient has signed informed consent. The inclusion criteria of participants in Cohort TEVAR are similar: a. patient scheduled for TEVAR because of thoracic aortic aneurysm or type B aortic dissection, b. patient has written informed consent.

The following exclusion criteria are applicable to both Cohort EVAR and TEVAR: a. emergent cases with ruptured or impending rupture aortic diseases, b. subjects with heavily calcified common femoral artery (more than 70% circumferential calcification).

#### Sample size

As no previous studies reported PCOs, we used the incidence of access site complications to estimate the sample size of the SWEET trial. According to previous studies, the incidence of access site complications in percutaneous EVAR was 7.64%, compared with 11.81% in cutdown EVAR (13). We estimated the number of patients needed in this non-inferiority trial with non-inferiority margin of 0.10 (bilateral α 5%, power 80%) by PASS 15.0 software, and 54 patients are required for percutaneous and cutdown EVAR group, respectively. To allow for 10% drop out, 60 patients will be recruited per group, i.e., 120 for Cohort EVAR.

Previous TEVAR studies lacked solid data in above aspects, however, the relevant data of transcatheter aortic valve implantation (TAVI) can be used for reference due to their similar surgical approach (15). The incidence of access site complications was 8.7% in percutaneous TAVI and 8.5% in cutdown TAVI (16). The sample size was calculated with non-inferiority margin of 0.15 (bilateral α 5 %, power 80 %), 45 patients are required for percutaneous and cutdown TEVAR group separately. Fifty patients will be recruited each group allowing for 10% drop out, and the number of subjects in Cohort TEVAR is 100. Therefore, the total sample size of the SWEET trial is 220. The whole calculation process can be found in Supplementary Tables S3, S4 (Supplementary Material).

### Intervention

In the percutaneous EVAR or TEVAR group, access is obtained through puncture of the common femoral artery deployment of two ProGlide devices (Abbott Vascular, Santa Clara, Calif) using a Preclose technique. The technique consists of deploying the needles of the first ProGlide device 30° medially or laterally from the midline. The second ProGlide needles are then deployed vertically from the first device, and then 16Fr sheath is inserted in the percutaneous access in EVAR and 18Fr sheath is used in TEVAR. When endovascular aortic repair is finished, the pretied knot and sutures of both devices are tightened and form knots by using the closure device remotely to achieve hemostasis. Pressuring and bandaging the puncture site gently also help stop bleeding. After percutaneous closure, it is necessary to observe whether the distal artery pulse well.

In the cutdown EVAR or TEVAR group, a 5-cm longitudinal incision is positioned in the groin area and common femoral artery is controlled by vessel loops. Femoral access is obtained through puncture under direct vision. After endovascular repair, the femoral artery is repaired thoroughly by a running 6-0 prolene suture. The subcutaneous tissue and skin are then sutured in standard fashion layer by layer.

### Assignment of intervention

**Figure 1:**
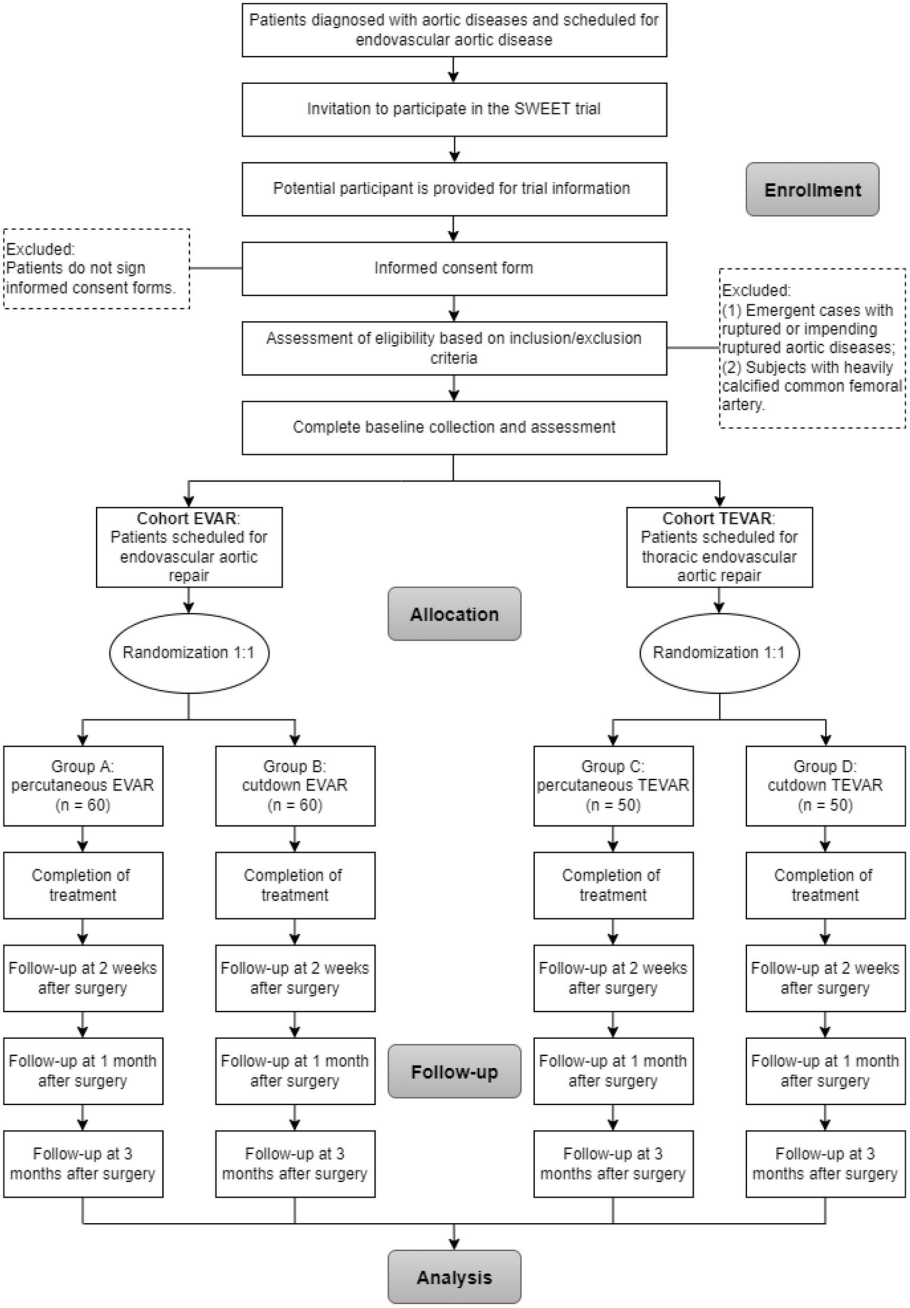
Flow of participants randomly assigned to percutaneous groups and cutdown groups.

Cohort EVAR and TEVAR in the SWEET trial are designed as a parallel randomized controlled, single-blind, single-center non-inferiority trial in a 1:1 ratio. The flow diagram for the study is outlined in Figure 1. After eligibility screening, eligible subjects in the two cohorts will be randomly allocated to either intervention groups receiving percutaneous access endovascular aortic repair or control groups receiving cutdown access endovascular aortic repair separately. As for patients who require brachial artery access in Cohort TEVAR, the assignment will be based on their femoral artery access, independent of the approach for upper extremity access.

Block randomization stratified by age and body mass index (BMI) using permuted blocks of random sizes will be performed with 1:1 allocation in both cohorts. Randomization sequence is generated by SPSS 26.0 statistical software by a biostatistician. The block size will be concealed until the primary endpoints are analyzed. An independent research coordinator will be responsible for keeping the sequentially numbered allocation sequence list, and will inform the surgeons about the assigned access procedure prior to intervention. To avoid performance bias, every surgeon must have had 10 or more ProGlide procedures, and experience of at least 20 surgical cutdowns to expose the access artery is also needed.

Obviously, trial participants and operating team cannot be blinded to the allocation. The data will be entered into the computer in separate tables by employees outside the research team so that the analysts can analyze data without knowing the allocation information. Therefore, only data analysts are blinded in this study.

### Outcome measures

The endpoints or composites in the SWEET trial are classified as ClinROs and PCOs. Cohort EVAR and TEVAR share the same primary and secondary endpoints. Primary and secondary outcomes will be assessed at 2 weeks, 1 month, and 3 months after surgery. An overview of the clinical outcome measures based on the SPIRIT recommendations is provided in Table 1.

**Table 1:** SPIRIT schedule for the SWEET randomized controlled trial.

	**Pre-study**		**Study visit**	**Follow-up**		
	**Enrollment**	**Baseline/** **Allocation**	**Treatment**	**2-week** **after surgery**	**1-month after surgery**	**3-month after surgery**
**Timepoint**	**-T1**	**0**	**T1**	**T2**	**T4**	**T5**
Enrollment						
Eligibility screen	**×**					
Informed consent	**×**					
Clinical interviews		**×**				
Allocation		**×**				
Interventions			**×**			
Assessments						
*Primary ClinRO endpoint*						
Access-related complications			**×**	**×**	**×**	
Access site infection			**×**	**×**	**×**	
Bleeding/ hematoma			**×**	**×**	**×**	
Access-related arterial injury			**×**	**×**	**×**	
Femoral artery occlusion			**×**	**×**	**×**	
Pseudoaneurysm			**×**	**×**	**×**	
Lymphorrhagia/seroma			**×**	**×**	**×**	
Access-related nerve injury			**×**	**×**	**×**	
Wound dehiscence			**×**	**×**	**×**	
*Primary PCO endpoint*						
Time back to normal life/work				**×**	**×**	**×**
*Secondary ClinRO endpoints*						
Operative time			**×**			
Length of hospital stay			**×**	**×**	**×**	
30-day limb graft occlusion			**×**	**×**	**×**	
30-day overall complications			**×**	**×**	**×**	
30-day mortality			**×**	**×**	**×**	
*Secondary PCO endpoints*						
Quality of life scores				**×**	**×**	
Duration of access-related pain				**×**	**×**	**×**

#### Primary outcomes

The primary ClinRO endpoint is access-related complications assessed in-hospital, 2 weeks and 1 month after surgery. The definition of access-related complications includes

- Access-site infection: inflammation of the groin presenting redness, swelling or exudation and requiring consecutive oral or intravenous antibiotics therapy.
- Bleeding/hematoma: fresh oozing blood or old blood stains seen on wound dressing/blood accumulation around the access site.
- Access-related arterial injury: arterial injuries requiring endarterectomy or patching due to the access technique.
- Femoral artery occlusion: femoral artery thrombosis presenting as distal artery pulse poorly requiring thrombectomy.
- Pseudoaneurysm: false aneurysm at the site of arterial injury presenting as a painful and pulsatile mass.
- Lymphorrhagia/seroma: swelling at access site caused by damage to lymphatic duct during access obtaining.
- Access-related nerve injury: nerve injuries presenting as persistent paresthesia of the thigh due to the access technique.
- Wound dehiscence: a partial or complete separation of previously close access wound edges.

The primary PCO endpoint is time (days) back to normal life/work assessed at 2 weeks by telephone interview and checked at 1 month and 3 months in outpatient clinics after surgery. To determine the outcomes that patients care most, we conducted a preliminary survey in 50 patients who received EVAR or TEVAR prior to our trial, and recovery time back to normal life or work represented the major PCO endpoint.

#### Secondary outcomes

The secondary ClinRO endpoints will be assessed in-hospital, 2 weeks and 1 month postoperatively, involving

- Operative time (minutes): defined as duration of whole EVAR or TEVAR procedure.
- Length of hospital stay (days): defined as the period of time a patient remains in hospital.
- 30-day limb graft occlusion: defined as a complete limb occlusion regardless of symptoms or lumen stenosis of more than 50% detected by image examination within 30 days postoperatively (17).
- 30-day overall complications: defined as all systemic or local complications within 30 days postoperatively, whether related to the access or not.
- 30-day mortality: defined as all-cause deaths occurring in the intervention population within 30 days postoperatively.

The secondary PCO endpoints involve quality of life scores and duration of access-related pain, which will be assessed simultaneously with the primary PCO endpoint.

- Quality of life scores: participants' perception of physical and mental health form various aspects over time, scored with European Quality of Life 5 Dimensions (EQ-5D) questionnaire.
- Duration of access-related pain (days): length of participants' unpleasant sensory and emotional experience associated with access wound.

#### Withdraw and dropout

Patients who agreed to participate in the trial can quit the study at any time for any reason without any consequences. After withdrawal, the subjects will not be replaced by others and their randomization number will not be re-used. Subjects are considered as dropout if they are lost to follow-up within 1-month postoperatively or they withdraw from the study.

### Data collection and management

#### Data collection methods

Baseline data required are shown in Supplementary Table S5 (Supplementary Material). Age, sex, BMI, comorbidity etc., should be collected before assignment to achieve maximum balance between groups. Anatomical characteristics of aortic aneurysm, aortic dissection and access artery that have potential impact on treatment success and prognosis should also be routinely measured by the operating team with computed tomography angiography (CTA). In particular, the heavily calcified femoral artery, defined as an estimated over 50% area of calcification in the superficial surface, needs more attention of investigator (7). So does the severe tortuous iliac artery, which is defined as any portion of iliac artery with tortuous angle more than 90° so that visually doubled or more on a single slice of axial CTA (18).

All patients after surgery will be examined for access-related complications during the everyday ward rounds and dressing changes. After discharge, the remaining primary and secondary outcomes can be gotten by telephone interviews and outpatient re-examination over several months. Quality of life scores will be quantified by EQ-5D questionnaire during follow-up, which reduces the variability in life quality assessment by various researchers. The EQ-5D questionnaire evaluates overall quality of life from 5 dimensions of mobility, self-care, usual activities, pain/discomfort, anxiety/depression, with each dimension ranging level 1 to 5. And its reliability and validity have been verified in many studies (19, 20).

#### Data management and confidentiality

In this trial, all data will be entered into the computer and several databases will be established. Modifications to data of the database will be documented. Moreover, data management personnel will use the mobile hard disk to back up the data of the databases once a month. Statisticians and supervisors will conduct regular data verification.

All written materials concerning to the study, including informed consent, medical history, surgical records, etc., will be securely stored in file cabinets. All random assignment, data collection, and follow-up management containing participant information will be conducted in the form of a web spreadsheet, which can only be accessed and edited with specific permissions. Participants' information will not be disclosed outside of this study without their written consent.

### Statistical methods

#### Analysis population

As Cohort EVAR and Cohort TEVAR were powered and randomized separately, the statistical analysis of both cohorts is planned to be reported separately. The analysis populations of this trial involve modified intention-to-treat (ITT) and per-protocol (PP) populations. The modified ITT is determined after randomization and when the patient started endovascular aortic repair, hence all patients who indeed receive endovascular aortic repair are involved in the primary analysis within the respective access group as originally allocated. In this trial, the modified ITT will be the main analysis set for the summary of both ClinROs and PCOs data. Both ITT and PP analysis are required for test of non-inferiority, and PP analysis serves as a sensitivity analysis. The non-inferiority margin was predetermined at 10%, and the non-inferiority test will be evaluated as a two-sided test at alpha = 0.05. When non-inferiority is reached, ITT is further tested for superiority.

#### Analysis of primary and secondary outcomes

Generalized linear model (GLM) will be used to compare continuous primary and secondary endpoints, and further adjustment for age, gender, BMI and femoral artery calcification will be performed by multivariate GLM analysis. Logistic regression using generalized estimating equations (GEE) will be applied to compare categorical primary and secondary endpoints. Using multivariate GEE models, the subsequent analysis will be adjusted for age, gender, BMI and femoral artery calcification. The access-related complications are counted and analyzed by the number of femoral accesses instead of the number of patients.

To address heterogeneity among study population, pre-specified subgroup analyses will be performed in the following populations: heavily calcified femoral artery vs. lightly calcified femoral artery, tortuous iliac artery vs. non-tortuous iliac artery, obesity or overweight vs. normal weight, smoking vs. none, elderly or octogenarian vs. younger population.

#### Handing of missing data

Missing data for baseline covariates will be addressed by multiple imputation in overall adjusted analyses, but those patients will not be included in the corresponding subgroup analysis.

### Monitoring

#### Data monitoring

Data Monitoring Committee (DMC), completely independent of the research team, will monitor the validity of data through reviewing interim analysis related to primary outcomes. And the interim analysis would be conducted by an independent statistician after 50% participants have been randomly assigned and completed a 3-month follow-up. The DMC will also make recommendations for the amendments of the study protocol according to the results of interim analysis.

#### Harms

Adverse events are defined as any unfavorable and unintended experience happening to participants during hospitalization and follow-up, whether they are considered to be related to the intervention or not. The presence of underlying disease at enrollment will not be reported as an adverse event, but any increase in the severity of the underlying disease will be considered an adverse event. Details of all adverse events reported voluntarily by participants or observed by investigators will be recorded on the case record forms, such as start date, end date, action taken, results etc.

Any event leading to death, prolonged or renewed hospitalization, disability or permanent damage could be described as a serious adverse event, which should be reported to the ethics committee timely. The principal investigator is required to conduct periodic cumulative reviews of all adverse events and, if necessary, convene meetings to assess the risks and benefits of the study.

#### Auditing

The DMC initial meeting will be held early stage of study, and the agenda contains familiarizing study background, reviewing study protocol, and setting a deadline of interim analysis report etc. Every 6 months, DMC will review enrollment data, adverse events data, validity and completeness of study data with unlimited access. If necessary, the DMC may request additional analysis beyond the interim analysis or an unscheduled security meeting to further understand the efficacy and safety of the trial.

### Ethics considerations and dissemination

#### Ethic approval

Ethics approval has been obtained from the Ethics Committee on Biomedical Research, West China Hospital of Sichuan University (approval number: 2021-1316) on 8th November 2021. Informed consent will be obtained from all participants, and the trial will be conducted in compliance with the Declaration of Helsinki and other regulations.

#### Protocol amendments

The protocol of SWEET trial may be amended during the progress of the trial, and any major amendments will be notified to the accredited medical research ethics committee and competent authority. Any revision in the informed consent forms will also be updated to the patients. Major amendments are defined as any change to the protocol that is likely to affect the conduct or management of the trial, safety of the patients or intervention details. Potential major amendments may include sample size adjustment based on actual clinical outcome difference between two groups, newly added outcomes and adjustment in statistical methods.

#### Ancillary and post-trial care

Participants will be compensated 2,000 yuan once primary wound adverse events occur. The primary wound adverse events are defined as access-site infection, bleeding/hematoma, access-related arterial injury, femoral artery occlusion, pseudoaneurysm, lymphorrhagia/seroma, access-related nerve injury and wound dehiscence during postoperative care in hospital or follow-up. China Postdoctoral Science Foundation will be responsible for the compensation.

#### Dissemination policy

The study has been registered in a public trial registry (www.chictr.org.cn). At the end of the SWEET trial, the principal investigator will write a summary concerning the main results and present it at annual congress or forum of vascular surgery in China. Simultaneously, related articles will be prepared for publication in an international authoritative journal. After approval by the principal investigator, all abstracts and publications concerning the primary and secondary outcomes from the trial could be submitted.

## Discussion

With the accelerated development of minimally invasive technology, the treatment for aortic diseases has undoubtedly entered the era of endovascular therapy. In line with the newest ESVS clinical guidelines, EVAR or TEVAR is the first-line option for aortic diseases with appropriate anatomy (3, 4). However, there is no consensus in vascular surgery community regarding the choice of access in endovascular aortic repair. In Sweden, 42% of all EVAR used percutaneous access in 2013, while 21% still used cutdown access simultaneously (21). The lack of high-quality evidence was the main reason for this phenomenon.

Previous cohort studies comparing percutaneous vs. cutdown EVAR or TEVAR demonstrated that percutaneous access had better outcomes on access site infection, wound healing and lymphorrhagia/seroma, while performed worse on pseudoaneurysm (8). However, those cohort studies analyzed patients from different periods with unequal treatment protocol and few studies reported standard deviation (SD) of continuous outcomes. Even though previous four RCTs compared percutaneous and cutdown access, their level of evidence is not high enough due to their outcomes measures, selection of reported results and inadequate sample size (9–12). Our trial uses access-related complications as the composite primary ClinRO endpoint, which contains infection, bleeding/hematoma, arterial injury, artery occlusion, pseudoaneurysm, lymphorrhagia/seroma, nerve injury, wound dehiscence. And this makes the primary outcome measures more statistically and clinically representative.

Patient-centered experience has been overlooked and rarely reported in studies, let alone as primary outcome. Uhlmann et al. evaluated access-related pain postoperatively by visual analog scale (VAS) and reported percutaneous EVAR did better in this aspect, however, solid data reflecting the quality of life and patient-centered experience were still lacking (10). Neither did the PiERO trial (9). PCO endpoints in the SWEET trial includes recovery time back to normal life, quality of life scores quantified by EQ-5D questionnaire and duration of access-related pain, which will fill in the blank.

Calcified femoral artery, tortuous iliac artery, obesity and inguinal scar were considered as risk factors for failure of percutaneous access EVAR (7, 22–24). Conversely, some researchers found that obesity and calcified femoral artery had no significant impact on the procedure success (25, 26). All these controversies were based on single-center experience or retrospective studies. The PEVAR trial stringently exclude patients with the any risk factor to ensure a highly homogeneous study population, but it also limited real-world applicability of the results (11). As for remaining trials, although selection criteria were wider, screening of participants were still limited by femoral artery calcification, previous femoral artery surgery, obesity and other conditions (9, 10). Our trial further broadens the selection criteria and plans pre-specified subgroup analysis based on population who break through these limitations. According to the results of the subgroup analysis, we hope to explore which part of the population would be suitable for and benefit from percutaneous endovascular aortic repair.

The choice of either percutaneous or cutdown access may not greatly affect the success of EVAR or TEVAR procedures, but can influence the quality of life and patient-centered experience. Given the very low evidence for ClinROs and few data for PCOs, comparison of the percutaneous vs. cutdown access EVAR and TEVAR is essential for both patient-centered care and clinical decision making in endovascular aortic repair.

## Ethics statement

The studies involving human participants were reviewed and approved by Ethics Committee on Biomedical Research, West China Hospital of Sichuan University. The patients/participants provided their written informed consent to participate in this study.

## Author contributions

YZ and JW contributed to the conception and design of the trial and drafted the manuscript. JW, JZ, and DY will recruit and screen the participants. YZ, JW, CW, TW, and BH will participate in data collection and analysis. JZ, DY, TW, and BH provided supervision support. All authors contributed to the critical revisions and final approval of the manuscript.

## Funding

The study is funded by the 69th batch of general support from China Postdoctoral Science Foundation (2021M692284). The funders have no role in study design, data collection, management and analysis, and decision to publish of the manuscript.

## Conflict of interest

The authors declare that the research was conducted in the absence of any commercial or financial relationships that could be construed as a potential conflict of interest.

## Publisher's note

All claims expressed in this article are solely those of the authors and do not necessarily represent those of their affiliated organizations, or those of the publisher, the editors and the reviewers. Any product that may be evaluated in this article, or claim that may be made by its manufacturer, is not guaranteed or endorsed by the publisher.
