# Supplementary material for: The effect of percutaneouS vs. cutdoWn accEss in patients after Endovascular aorTic repair (SWEET): Study protocol for a single-blind, single-center, randomized controlled trial
Source: Front Cardiovasc Med. 2022 Aug 19;9:966251. doi: 10.3389/fcvm.2022.966251 (PMC9437429; doi:10.3389/fcvm.2022.966251)
Supplement: Supplementary file 1 [file Data_Sheet_1.docx]

Supplementary Material

**Table S1**. SPIRIT 2013 checklist for the SWEET RCT: Recommended items to address in a clinical trial protocol and related documents

| Section/item | Item  No | Description | Addresses on page number |
| --- | --- | --- | --- |
| Administrative information | | |  |
| Title | 1 | Descriptive title identifying the study design, population, interventions, and, if applicable, trial acronym | 1 |
| Trial registration | 2a | Trial identifier and registry name. If not yet registered, name of intended registry | 1 |
|  | 2b | All items from the World Health Organization Trial Registration Data Set | Yes, chictr.org.cn |
| Protocol version | 3 | Date and version identifier | 1 |
| Funding | 4 | Sources and types of financial, material, and other support | 9 |
| Roles and responsibilities | 5a | Names, affiliations, and roles of protocol contributors | 1, 9 |
|  | 5b | Name and contact information for the trial sponsor | 9 |
|  | 5c | Role of study sponsor and funders, if any, in study design; collection, management, analysis, and interpretation of data; writing of the report; and the decision to submit the report for publication, including whether they will have ultimate authority over any of these activities | 9 |
|  | 5d | Composition, roles, and responsibilities of the coordinating centre, steering committee, endpoint adjudication committee, data management team, and other individuals or groups overseeing the trial, if applicable (see Item 21a for data monitoring committee) | 7 |
| Introduction | | |  |
| Background and rationale | 6a | Description of research question and justification for undertaking the trial, including summary of relevant studies (published and unpublished) examining benefits and harms for each intervention | 2 |
|  | 6b | Explanation for choice of comparators | 2 |
| Objectives | 7 | Specific objectives or hypotheses | 2 |
| Trial design | 8 | Description of trial design including type of trial (eg, parallel group, crossover, factorial, single group), allocation ratio, and framework (eg, superiority, equivalence, noninferiority, exploratory) | 2 |
| Methods: Participants, interventions, and outcomes | | |  |
| Study setting | 9 | Description of study settings (eg, community clinic, academic hospital) and list of countries where data will be collected. Reference to where list of study sites can be obtained | 2 |
| Eligibility criteria | 10 | Inclusion and exclusion criteria for participants. If applicable, eligibility criteria for study centres and individuals who will perform the interventions (eg, surgeons, psychotherapists) | 3, 4 |
| Interventions | 11a | Interventions for each group with sufficient detail to allow replication, including how and when they will be administered | 3, 4 |
|  | 11b | Criteria for discontinuing or modifying allocated interventions for a given trial participant (eg, drug dose change in response to harms, participant request, or improving/worsening disease) | 2, 5 |
|  | 11c | Strategies to improve adherence to intervention protocols, and any procedures for monitoring adherence (eg, drug tablet return, laboratory tests) | 2, 4, 5 |
|  | 11d | Relevant concomitant care and interventions that are permitted or prohibited during the trial | n/a |
| Outcomes | 12 | Primary, secondary, and other outcomes, including the specific measurement variable (eg, systolic blood pressure), analysis metric (eg, change from baseline, final value, time to event), method of aggregation (eg, median, proportion), and time point for each outcome. Explanation of the clinical relevance of chosen efficacy and harm outcomes is strongly recommended | 4, 5 and Table 1 |
| Participant timeline | 13 | Time schedule of enrolment, interventions (including any run-ins and washouts), assessments, and visits for participants. A schematic diagram is highly recommended (see fig 1) | 2, 3, 4, Figure 1 and Table 1 |
| Sample size | 14 | Estimated number of participants needed to achieve study objectives and how it was determined, including clinical and statistical assumptions supporting any sample size calculations | 3 |
| Recruitment | 15 | Strategies for achieving adequate participant enrolment to reach target sample size | 3 |
| Methods: Assignment of interventions (for controlled trials) | | |  |
| Allocation: |  |  |  |
| Sequence generation | 16a | Method of generating the allocation sequence (eg, computer-generated random numbers), and list of any factors for stratification. To reduce predictability of a random sequence, details of any planned restriction (eg, blocking) should be provided in a separate document that is unavailable to those who enrol participants or assign interventions | 4 |
| Allocation concealment mechanism | 16b | Mechanism of implementing the allocation sequence (eg, central telephone; sequentially numbered, opaque, sealed envelopes), describing any steps to conceal the sequence until interventions are assigned | 4 |
| Implementation | 16c | Who will generate the allocation sequence, who will enrol participants, and who will assign participants to interventions | 4 |
| Blinding (masking) | 17a | Who will be blinded after assignment to interventions (eg, trial participants, care providers, outcome assessors, data analysts) and how | 4 |
|  | 17b | If blinded, circumstances under which unblinding is permissible and procedure for revealing a participant’s allocated intervention during the trial | n/a |
| Methods: Data collection, management, and analysis | | |  |
| Data collection methods | 18a | Plans for assessment and collection of outcome, baseline, and other trial data, including any related processes to promote data quality (eg, duplicate measurements, training of assessors) and a description of study instruments (eg, questionnaires, laboratory tests) along with their reliability and validity, if known. Reference to where data collection forms can be found, if not in the protocol | 6 |
|  | 18b | Plans to promote participant retention and complete follow-up, including list of any outcome data to be collected for participants who discontinue or deviate from intervention protocols | 2 |
| Data management | 19 | Plans for data entry, coding, security, and storage, including any related processes to promote data quality (eg, double data entry; range checks for data values). Reference to where details of data management procedures can be found, if not in the protocol | 6 |
| Statistical methods | 20a | Statistical methods for analysing primary and secondary outcomes. Reference to where other details of the statistical analysis plan can be found, if not in the protocol | 6, 7 |
|  | 20b | Methods for any additional analyses (eg, subgroup and adjusted analyses) | 7 |
|  | 20c | Definition of analysis population relating to protocol non-adherence (eg, as randomised analysis), and any statistical methods to handle missing data (eg, multiple imputation) | 6, 7 |
| Methods: Monitoring | | |  |
| Data monitoring | 21a | Composition of data monitoring committee (DMC); summary of its role and reporting structure; statement of whether it is independent from the sponsor and competing interests; and reference to where further details about its charter can be found, if not in the protocol. Alternatively, an explanation of why a DMC is not needed | 7 |
|  | 21b | Description of any interim analyses and stopping guidelines, including who will have access to these interim results and make the final decision to terminate the trial | 7 |
| Harms | 22 | Plans for collecting, assessing, reporting, and managing solicited and spontaneously reported adverse events and other unintended effects of trial interventions or trial conduct | 7 |
| Auditing | 23 | Frequency and procedures for auditing trial conduct, if any, and whether the process will be independent from investigators and the sponsor | 7 |
| Ethics and dissemination | | |  |
| Research ethics approval | 24 | Plans for seeking research ethics committee/institutional review board (REC/IRB) approval | 8 |
| Protocol amendments | 25 | Plans for communicating important protocol modifications (eg, changes to eligibility criteria, outcomes, analyses) to relevant parties (eg, investigators, REC/IRBs, trial participants, trial registries, journals, regulators) | 8 |
| Consent or assent | 26a | Who will obtain informed consent or assent from potential trial participants or authorised surrogates, and how (see Item 32) | 2 |
|  | 26b | Additional consent provisions for collection and use of participant data and biological specimens in ancillary studies, if applicable | n/a |
| Confidentiality | 27 | How personal information about potential and enrolled participants will be collected, shared, and maintained in order to protect confidentiality before, during, and after the trial | 7 |
| Declaration of interests | 28 | Financial and other competing interests for principal investigators for the overall trial and each study site | 9 |
| Access to data | 29 | Statement of who will have access to the final trial dataset, and disclosure of contractual agreements that limit such access for investigators | 7, 8 |
| Ancillary and post-trial care | 30 | Provisions, if any, for ancillary and post-trial care, and for compensation to those who suffer harm from trial participation | 8 |
| Dissemination policy | 31a | Plans for investigators and sponsor to communicate trial results to participants, healthcare professionals, the public, and other relevant groups (eg, via publication, reporting in results databases, or other data sharing arrangements), including any publication restrictions | 8 |
|  | 31b | Authorship eligibility guidelines and any intended use of professional writers | 9 |
|  | 31c | Plans, if any, for granting public access to the full protocol, participant-level dataset, and statistical code | 8 |
| Appendices | | |  |
| Informed consent materials | 32 | Model consent form and other related documentation given to participants and authorised surrogates | Available on request |
| Biological specimens | 33 | Plans for collection, laboratory evaluation, and storage of biological specimens for genetic or molecular analysis in the current trial and for future use in ancillary studies, if applicable | n/a |

**Table S2**. Overview of the study derived from the PICO framework

| **Criteria** | **Assessments** |
| --- | --- |
| **P:**  **Population** | ***Inclusion criteria in Cohort EVAR:***  (1) Patient scheduled for EVAR because of abdominal aorto-iliac artery aneurysm or dissection;  (2) Patient has signed informed consent.  ***Inclusion criteria in Cohort TEVAR:***  (1) Patient scheduled for TEVAR because of thoracic aortic aneurysm or type B aortic dissection;  (2) Patient has written informed consent.  ***Exclusion criteria in both Cohort EVAR and Cohort TEVAR:***  (1) Emergent cases with ruptured or impending rupture aortic diseases;  (2) Subjects with heavily calcified common femoral artery (more than 70% circumferential calcification). |
| **I:**  **Intervention** | - Percutaneous access EVAR - Percutaneous access TEVAR |
| **C:**  **Control** | - Cutdown access EVAR - Cutdown access TEVAR |
| **O:**  **Outcome** | ***Primary outcomes:***  ***Primary clinician-reported outcomes (ClinROs):***  Access-related complications   - Access-site infection - Bleeding/hematoma - Access-related arterial injury - Femoral artery occlusion - Pseudoaneurysm - Lymphorrhagia/seroma - Access-related nerve injury - Wound dehiscence   ***Primary patient-centered outcomes (PCOs):***  Time back to normal life/work  ***Secondary outcomes:***  ***Secondary clinician-reported outcomes (ClinROs):***   - Operative time - Length of hospital stay - 30-day limb graft occlusion - 30-day overall complications - 30-day mortality   ***Secondary patient-centered outcomes (PCOs):***   - Quality of life scores - Duration of access-related pain |

**Supplement S3. Sample size calculation for EVAR cohort**

**Numeric Results for Non-Inferiority Tests for the Difference Between Two Proportions**

Test Statistic: Z-Test with Unpooled Variance

H0: P1 - P2 ≥ D0 vs. H1: P1 - P2 = D1 < D0.

**Target Actual Target Actual Ref. P1|H0 P1|H1 NI Diff Diff**

**Power Power* N1 N2 N R R P2 P1.0 P1.1 D0 D1 Alpha**

0.80 0.80131 54 54 108 1.00 1.00 0.1181 0.2181 0.0764 0.1000 -0.0417 0.050

0.80 0.80707 30 30 60 1.00 1.00 0.1181 0.2681 0.0764 0.1500 -0.0417 0.050

0.80 0.80939 19 19 38 1.00 1.00 0.1181 0.3181 0.0764 0.2000 -0.0417 0.050

* Power was computed using the normal approximation method.

**Report Definitions**

Target Power is the desired power value (or values) entered in the procedure. Power is the probability of

rejecting a false null hypothesis.

Actual Power is the power obtained in this scenario. Because N1 and N2 are discrete, this value is often

(slightly) larger than the target power.

N1 and N2 are the number of items sampled from each population.

N is the total sample size, N1 + N2.

Target R is the desired ratio (or ratios) of R entered in the procedure. R is the ratio of N2 to N1, so that

N2 = R × N1.

Actual R is the value for R obtained in this scenario. Because N1 and N2 are discrete, this value is sometimes

slightly different than the target R.

P2 is the proportion for Group 2. This is the standard, reference, or control group.

P1 is the treatment or experimental group proportion. P1.0 is the largest treatment-group response rate that

still yields a non-inferiority conclusion. P1.1 is the proportion for Group 1 at which power and sample size

calculations are made.

D0 is the non-inferiority margin. It is the difference P1 - P2, assuming H0. D1 is the difference P1 - P2

assumed for power and sample size calculations.

Alpha is the probability of rejecting a true null hypothesis.

**Summary Statements**

Sample sizes of 54 in Group 1 and 54 in Group 2 achieve 80.131% power to detect a non-inferiority margin difference between the group proportions of 0.1000. The reference group proportion is 0.1181. The treatment group proportion is assumed to be 0.2181 under the null hypothesis of inferiority. The power was computed for the case when the actual treatment group proportion is 0.0764. The test statistic used is the one-sided Z test (unpooled). The significance level of the test is 0.050.

**Dropout-Inflated Sample Size**

**Dropout-Inflated Expected**

**Enrollment Number of**

**──── Sample Size ──── ──── Sample Size ──── ───── Dropouts ─────**

**Dropout Rate N1 N2 N N1' N2' N' D1 D2 D**

20% 54 54 108 68 68 136 14 14 28

20% 30 30 60 38 38 76 8 8 16

20% 19 19 38 24 24 48 5 5 10

**Definitions**

Dropout Rate (DR) is the percentage of subjects (or items) that are expected to be lost at random during the

course of the study and for whom no response data will be collected (i.e. will be treated as "missing").

N1, N2, and N are the evaluable sample sizes at which power is computed. If N1 and N2 subjects are

evaluated out of the N1' and N2' subjects that are enrolled in the study, the design will achieve the stated

power.

N1', N2', and N' are the number of subjects that should be enrolled in the study in order to end up with N1, N2,

and N evaluable subjects, based on the assumed dropout rate. After solving for N1 and N2, N1' and N2'

are calculated by inflating N1 and N2 using the formulas N1' = N1 / (1 - DR) and N2' = N2 / (1 - DR), with

N1' and N2' always rounded up. (See Julious, S.A. (2010) pages 52-53, or Chow, S.C., Shao, J., and

Wang, H. (2008) pages 39-40.)

D1, D2, and D are the expected number of dropouts. D1 = N1' - N1, D2 = N2' - N2, and D = D1 + D2.

**Supplement S4. Sample size calculation for TEVAR cohort**

**Numeric Results for Non-Inferiority Tests for the Difference Between Two Proportions**

Test Statistic: Z-Test with Unpooled Variance

H0: P1 - P2 ≥ D0 vs. H1: P1 - P2 = D1 < D0.

**Target Actual Target Actual Ref. P1|H0 P1|H1 NI Diff Diff**

**Power Power* N1 N2 N R R P2 P1.0 P1.1 D0 D1 Alpha**

0.80 0.80273 102 102 204 1.00 1.00 0.0850 0.1850 0.0870 0.1000 0.0020 0.050

0.80 0.80487 45 45 90 1.00 1.00 0.0850 0.2350 0.0870 0.1500 0.0020 0.050

0.80 0.80291 25 25 50 1.00 1.00 0.0850 0.2850 0.0870 0.2000 0.0020 0.050

* Power was computed using the normal approximation method.

**Report Definitions**

Target Power is the desired power value (or values) entered in the procedure. Power is the probability of

rejecting a false null hypothesis.

Actual Power is the power obtained in this scenario. Because N1 and N2 are discrete, this value is often

(slightly) larger than the target power.

N1 and N2 are the number of items sampled from each population.

N is the total sample size, N1 + N2.

Target R is the desired ratio (or ratios) of R entered in the procedure. R is the ratio of N2 to N1, so that

N2 = R × N1.

Actual R is the value for R obtained in this scenario. Because N1 and N2 are discrete, this value is sometimes

slightly different than the target R.

P2 is the proportion for Group 2. This is the standard, reference, or control group.

P1 is the treatment or experimental group proportion. P1.0 is the largest treatment-group response rate that

still yields a non-inferiority conclusion. P1.1 is the proportion for Group 1 at which power and sample size

calculations are made.

D0 is the non-inferiority margin. It is the difference P1 - P2, assuming H0. D1 is the difference P1 - P2

assumed for power and sample size calculations.

Alpha is the probability of rejecting a true null hypothesis.

**Summary Statements**

Sample sizes of 102 in Group 1 and 102 in Group 2 achieve 80.273% power to detect a non-inferiority margin difference between the group proportions of 0.1000. The reference group proportion is 0.0850. The treatment group proportion is assumed to be 0.1850 under the null hypothesis of inferiority. The power was computed for the case when the actual treatment group proportion is 0.0870. The test statistic used is the one-sided Z test (unpooled). The significance level of the test is 0.050.

**Dropout-Inflated Sample Size**

**Dropout-Inflated Expected**

**Enrollment Number of**

**──── Sample Size ──── ──── Sample Size ──── ───── Dropouts ─────**

**Dropout Rate N1 N2 N N1' N2' N' D1 D2 D**

20% 102 102 204 128 128 256 26 26 52

20% 45 45 90 57 57 114 12 12 24

20% 25 25 50 32 32 64 7 7 14

**Definitions**

Dropout Rate (DR) is the percentage of subjects (or items) that are expected to be lost at random during the

course of the study and for whom no response data will be collected (i.e. will be treated as "missing").

N1, N2, and N are the evaluable sample sizes at which power is computed. If N1 and N2 subjects are

evaluated out of the N1' and N2' subjects that are enrolled in the study, the design will achieve the stated

power.

N1', N2', and N' are the number of subjects that should be enrolled in the study in order to end up with N1, N2,

and N evaluable subjects, based on the assumed dropout rate. After solving for N1 and N2, N1' and N2'

are calculated by inflating N1 and N2 using the formulas N1' = N1 / (1 - DR) and N2' = N2 / (1 - DR), with

N1' and N2' always rounded up. (See Julious, S.A. (2010) pages 52-53, or Chow, S.C., Shao, J., and

Wang, H. (2008) pages 39-40.)

D1, D2, and D are the expected number of dropouts. D1 = N1' - N1, D2 = N2' - N2, and D = D1 + D2.

**Table S5**. Required baseline data collection schedule.

|  | Pre-study | | Study visit |
| --- | --- | --- | --- |
|  | Enrollment | Baseline/  Allocation | Treatment |
| Timepoint | -T1 | 0 | T1 |
| Enrollment | **×** |  |  |
| Baseline Data |  |  |  |
| *Clinical interviews* |  |  |  |
| Age |  | **×** |  |
| Height |  | **×** |  |
| Weight |  | **×** |  |
| Body mass index (BMI) |  | **×** |  |
| Self-care ability |  | **×** |  |
| Length of medical history |  | **×** |  |
| Symptom |  | **×** |  |
| Smoking |  | **×** |  |
| Hypertension |  | **×** |  |
| Diabetes mellitus |  | **×** |  |
| Chronic obstructive pulmonary disease (COPD) |  | **×** |  |
| Coronary heart disease |  | **×** |  |
| Coronary intervention |  | **×** |  |
| Stroke |  | **×** |  |
| Heart function grade of New York Heart Association (NYHA) |  | **×** |  |
| Arrhythmia |  | **×** |  |
| Chronic kidney disease |  | **×** |  |
| Tumor |  | **×** |  |
| Infection |  | **×** |  |
| Marfan Syndrome |  | **×** |  |
| *Anatomic measurement of aortic aneurysm* |  |  |  |
| Aortic neck α angle |  | **×** |  |
| Aortic neck β angle |  | **×** |  |
| Aortic neck length |  | **×** |  |
| Aortic neck diameter |  | **×** |  |
| Aortic neck calcification |  | **×** |  |
| Aortic aneurysm diameter |  | **×** |  |
| Aortic aneurysm thrombus |  | **×** |  |
| *Anatomic measurement of aortic dissection* |  |  |  |
| Aortic arch type |  | **×** |  |
| Major axis of minimum diameter of descending aortic true lumen |  | **×** |  |
| Minor axis of minimum diameter of descending aortic true lumen |  | **×** |  |
| Major axis of maximum diameter of descending aortic false lumen |  | **×** |  |
| Minor axis of maximum diameter of descending aortic false lumen |  | **×** |  |
| Initial zone of dissection |  | **×** |  |
| Distal zone of dissection |  | **×** |  |
| Primary entry tear zone |  | **×** |  |
| Distal tear zones |  | **×** |  |
| Blood supply by left renal artery |  | **×** |  |
| Blood supply by right renal artery |  | **×** |  |
| Blood supply by superior mesenteric artery |  | **×** |  |
| Blood supply by celiac artery |  | **×** |  |
| Intervention of left subclavian artery |  |  | **×** |
| Intervention of visceral artery |  |  | **×** |
| Intervention of distal tear |  |  | **×** |
| Proximal diameter of the first stent |  |  | **×** |
| Distal diameter of the first stent |  |  | **×** |
| Proximal diameter of the second stent |  |  | **×** |
| Distal diameter of the second stent |  |  | **×** |
| *Anatomic measurement of access artery* |  |  |  |
| Subcutaneous thickness of left femoral artery |  | **×** |  |
| Subcutaneous thickness of right femoral artery |  | **×** |  |
| Calcification of left femoral artery |  | **×** |  |
| Calcification of right femoral artery |  | **×** |  |
| Diameter of left common femoral artery |  | **×** |  |
| Diameter of right common femoral artery |  | **×** |  |
| Maximum diameter of left iliac artery |  | **×** |  |
| Maximum diameter of right iliac artery |  | **×** |  |
| Minimum diameter of left iliac artery |  | **×** |  |
| Minimum diameter of right iliac artery |  | **×** |  |
| Tortuosity index of left iliac artery |  | **×** |  |
| Tortuosity index of right iliac artery |  | **×** |  |
| Diameter of left iliac artery sealing zone |  |  | **×** |
| Diameter of right iliac artery sealing zone |  |  | **×** |
| Seal length of left iliac artery sealing zone |  |  | **×** |
| Seal length of right iliac artery sealing zone |  |  | **×** |
| *Allocation* |  | **×** |  |
